# Hearing screening of Grade R learners in Qumbu: Referral rates and potential hearing loss

**DOI:** 10.4102/phcfm.v17i1.5092

**Published:** 2025-12-11

**Authors:** Antonette R. Pierce, Unati Stemela-Zali

**Affiliations:** 1 Department of Nursing and Public Health, Faculty of Health Sciences, University of Fort Hare, East London, South Africa; 2 Department of Natural and Rehabilitative Sciences, Faculty of Health Sciences, University of Fort Hare, East London, South Africa

**Keywords:** hearing screening, Grade R learners, Eastern Cape, referral rate, childhood hearing loss

## Abstract

**Background:**

Hearing loss in children has a profound impact on development. It negatively affects speech production, language acquisition, social interaction and early cognitive growth. These challenges influence emergent literacy if undetected and untreated.

**Aim:**

This study aimed to determine the referral rate and potential prevalence of hearing loss among Grade R learners in the Qumbu Circuit Management Centre (CMC).

**Setting:**

Nine primary schools located in Qumbu CMC, a rural area within the O.R. Tambo Inland District of the Eastern Cape province, South Africa.

**Methods:**

A quantitative, cross-sectional design was used. Out of 215 schools, 10 were conveniently selected using Slovin’s formula. From these, 259 Grade R learners aged 5–6 years were randomly selected to participate. Hearing screening followed the minimum standards for school-based screening set by the Professional Board for Speech, Language, and Hearing Professions in October 2018. Data were analysed using Statistical Package for Social Sciences (SPSS) version 27.

**Results:**

Screening outcomes revealed that 25.5% of the learners presented with ear-related conditions requiring referral for further diagnostic evaluation and management. Conditions on findings included wax impaction and otitis media.

**Conclusion:**

The study revealed a notable referral rate and potential prevalence of both temporary and permanent hearing loss among learners in Qumbu CMC. These results emphasise the importance of regular screening, early detection and timely intervention in underserved rural communities.

**Contribution:**

This study provides baseline data to guide future research and inform planning for hearing healthcare in the Eastern Cape.

## Introduction

It is noted that around 466 million people are living with a hearing loss, and 34 million are children worldwide.^1^ It is estimated that by 2030, this number will grow to more than 630 million, and more than 900 million by 2050.^1^ South Africa has a population of 10.6 million children, with an estimated 1.5 million aged 5 years to 14 years living with a hearing loss.^2^ One of the notable negative impacts of hearing loss is the inability to communicate with others, particularly in children, where it often leads to delayed spoken language development.^1^ The healthcare realities, such as the burden of diseases and poor social determinants of health, exacerbate the high risk for hearing impairment amongst people living in low- and middle-income countries like South Africa.^3^ These low- and middle-income countries constitute 80% of the world’s population and are home to two-thirds of individuals with hearing impairment.^4^ A study conducted in the Western Cape province on the prevalence of hearing and vision loss found that hearing loss is prevalent in at least 22 per 1000 preschool children in an underserved South African community.^5^ In the Eastern Cape province of South Africa, where this study was done, hearing healthcare is very limited, and one of the reasons is the shortage of audiology services in public health care facilities.^6^ This warrants the need for this study in Eastern Cape province.

Early detection of hearing loss is vital for communication development and school readiness.^7^ Screening primarily refers to the procedure of detecting healthy individuals who may present with a possible hearing impairment that may require further diagnostic evaluation.^8^ The Health Professions Council of South Africa (HPCSA), together with the World Health Organization (WHO), provides guidelines for hearing screening. Among the guidelines provided are the frequency of screening across multiple life stages, screening tools and the pass or fail criteria. This study followed these three guidelines by selecting the preschool age as one of the critical life stages for hearing screening, using tools like pure-tone audiometry (1000, 2000 and 4000 Hz at 20 dB HL), and adhering to the pass criteria guideline, which is a response to all tones in both ears, and referral if no response at any frequency.^1,9^

Further to these guidelines, Martin and Clark^10^ noted that for hearing screening to be beneficial, the process has to be specific to the target population and use a sensitive test battery. The screening must be able to identify those individuals who have failed the screening, indicating potential hearing loss, as well as those who have passed and have normal hearing function. Results of hearing screening in each ear are then compared (right and left ear); passing results suggest normal hearing, while failing results indicate the need for further diagnostic evaluation.^10^ The earlier a hearing impairment is identified and intervention provided, the higher the chances of reducing or minimising its negative effects, allowing children with hearing impairment the opportunity to develop to their full potential.^9,11,12^

Children exposed to these risk factors require ongoing surveillance and periodic hearing screening, as hearing loss can develop at any stage.^9^ Studies conducted in the Oliver Reginald Tambo District of the Eastern Cape confirmed the association between social determinants of health and human immunodeficiency virus (HIV) status and hearing loss in children.^13,14^ Another study confirmed late identification of hearing loss in children of the same region.^6^ The HPCSA.^9^ and the Joint Committee on Infant Hearing (JCIH)^15^ list the following as common risk factors: caregiver concern about hearing, speech, language or developmental delay; family history of permanent childhood hearing loss; Neonatal Intensive Care Unit (NICU) stay of more than 5 days; in utero infections (e.g. cytomegalovirus (CMV), rubella, syphilis); craniofacial anomalies; syndromes associated with hearing loss (e.g. Down, Usher); neurodegenerative disorders; postnatal infections (e.g. meningitis, measles); head trauma; ototoxic medications; recurrent or persistent otitis media with effusion for at least 3 months; and low birth weight or prematurity (< 1500 g or < 32 weeks). Children exposed to these risk factors require ongoing surveillance and periodic hearing screening, as hearing loss can develop at any stage.^9^ Ear diseases such as otitis media and hearing loss negatively impact children’s education, often delaying language development and affecting academic performance.^1^ Early identification and intervention are fundamental to reducing these impacts and improving educational outcomes in children with hearing loss.^1^ Preschool and school-based hearing screenings are considered most effective for early detection and management.^1^ Children from the Qumbu CMC are at risk of exposure to both low socio-economic environments and medical conditions, which could lead to hearing loss, and, therefore, conducting screening in this context was necessary.

In South Africa, there are few reports on hearing screening programmes, and the available reports are mostly from developed provinces such as the Western Cape,^16^ Gauteng^3,17^ and KwaZulu-Natal.^18^ A few programmes have also emerged in under-resourced provinces like Limpopo.^19^ These initiatives have been guided by the Integrated School Health Policy (ISHP) and the HPCSA’s minimum standards.^20^ However, developing and sustaining screening programmes in schools has proven challenging.^18^ The ISHP, reviewed in 2011, highlighted numerous barriers, including a lack of technical and human resources, financial constraints and transport issues.^18^ Although more work has been done since 2011, the study by Kgare and Joubert shows no change in the barriers to hearing screening programmes.^19^

The review of the ISHP conducted in 2012 aimed to address immediate health challenges of learners and stimulate their overall well-being throughout childhood and adolescence.^21^ The policy provided services to all school-going children, including pre-schoolers (Grade R), but excluded those who had completed Grade 12 or were not attending school in 2024, which revealed that the screening remains far below national targets, with significant variation across provinces, especially for Grade 1 learners (*n* = 21); research conducted by undergraduates from the University of KwaZulu-Natal in 2015 revealed that Grade 1 learners in urban Durban experienced common ear issues such as cerumen impaction, middle ear pathologies and possible hearing loss.^18^

Audiologists from the Eastern Cape Department of Education noted that the mass hearing screening conducted during ‘World Hearing Week’ in 2019 across various districts enabled early detection of hearing loss in Grade R learners.^22^ This project sparked an interest in this line of study and laid the groundwork for this quantitative cross-sectional study.

Early detection of hearing loss is critical for optimal communication development and school readiness. Although the WHO and HPCSA provide guidelines for hearing screening across developmental stages, implementation is limited in many parts of South Africa. This is especially true in predominantly rural and under-resourced provinces such as the Eastern Cape, where routine screening for preschool-aged children is rare and audiology services are scarce, particularly at primary health care and school levels.

While some screening programmes have been reported in the Western Cape, Gauteng and KwaZulu-Natal, the Eastern Cape still lacks sufficient published data. Phanguphangu et al. and Kgare et al. have published work in a similar context, but the focus was on social determinants of health in relation to hearing loss in children, HIV exposure and hearing loss in children and availability of screening resources in healthcare facilities. A mass hearing screening conducted in 2019 by provincial audiologists revealed a high prevalence of unidentified hearing problems among Grade R learners, emphasising the need for structured interventions in this region.^22^

Given the documented risk factors and healthcare challenges in the Eastern Cape, this study seeks to provide essential data to inform health policy and improve early hearing detection services for young children.^6^ It brings forth screening results conducted in a district on the outskirts, which exemplifies remote rural regions and foregrounds research opportunities for prevalence studies of hearing loss in a critical age group.

## Research methods and design

### Study design

A descriptive quantitative cross-sectional study design was used.

### Study setting

The study was carried out in primary schools under the Qumbu Circuit Management Centre (CMC), which includes 129 public primary schools. The Qumbu CMC falls within the jurisdiction of the Qumbu Satellite, located in the O.R. Tambo Inland District of the Eastern Cape province, South Africa. Qumbu is a predominantly rural area situated approximately 61 km north of Mthatha, comprising a total of 207 public primary schools. These are distributed between the Qumbu CMC (*n* = 129) and Tsolo (*n* = 78).

The Eastern Cape is among the provinces most affected by structural poverty, with rates estimated between 20% and 60%. Within the province, poverty levels are particularly high in Alfred Nzo (57.7%), O.R. Tambo (53.9%) and Joe Gqabi (49.8%).^16^ Qumbu was selected as the study site, as it represents the socio-economic and infrastructural challenges characteristic of rural and under-resourced communities in South Africa.

The study was carried out in school settings to ensure that the environment was familiar to learners. Hearing screening was conducted in a quiet classroom with reduced ambient noise, thereby minimising external interferences with the testing procedure.

### Study population and sampling strategy

The study population comprised 259 Grade R learners aged 5–6 years enrolled in 9 of the 129 public primary schools in Qumbu CMC. The 10th school did not participate, as the term for data collection at schools had lapsed. Learners were eligible if they were within the target age range, enrolled in Grade R at a public school in the district and if informed parental consent was obtained. Learners were excluded if they were outside the specified age range or if parental consent was not granted.

The sample size of the schools was calculated using Slovin’s formula,^23^ which indicated that a minimum of 10 schools would be sufficient when accepting a 20% – 30% error margin. This error margin was accepted because the aim of the study was not to generalise the results but to make a case for the potential prevalence of hearing loss in the Qumbu CMC.

The total number of learners enrolled in Grade R at these 10 schools was 481. Using the Slovan’s formula with an error margin of 5%, a total of 219 learners would be sufficient. A pleasant surprise of 259 learners ultimately participated in the study, reflecting voluntary participation and positive parental consent. This participant’s turnout strengthened the results of the study.

Before data for the main study were collected, a pilot study preceded. A pilot study was conducted in two schools that met the inclusion criteria for schools but would not be included in the main study. The objective of the pilot study was to assess the validity and reliability of the research tools and instruments. The results identified the need to distribute the consent forms in advance, as same-day completion was not giving adequate time for parents to fully engage with the content of the forms before deciding whether to participate or not. Recommendations from the pilot were then implemented for the main study.

### Data collection

Three research tools were utilised to collect data. These were, namely, the screening audiogram and the hearing screening equipment. Hearing assessments were conducted in quiet classroom settings to minimise ambient noise and ensure accurate measurement. Clinical screening equipment, including an otoscope, screening tympanometer and screening audiometer, was used to perform the evaluations. Data gathering was done using a screening audiogram to record hearing screening results. The audiogram was based on a hearing screening form developed by the Eastern Cape Department of Education, aligning with local screening protocols. Learners’ results were recorded directly on an audiogram, using standard symbols, to indicate right- and left-ear thresholds across frequencies and intensities.^10^

Screening equipment included a Welch-Allyn Mini 3000 otoscope, a GSI 33 hand-held screening tympanometer and a GSI 17 audiometer (HASS Group; Grason-Stadler). Equipment maintenance and calibration adhered to South African Bureau of Standards (SABS) 0154-1/2 and 0182 standards, in line with HPCSA Minimum Standards for Hearing Screening in Schools.^9^ The hearing screening was conducted by the researcher, a qualified audiologist, in quiet rooms on school premises. Ambient noise levels were monitored using the Decibel X application on a Samsung Galaxy S21 Ultra smartphone to measure A-weighted decibels (dBA). Although not a substitute for calibrated ANSI/IEC sound level meters, smartphone-based apps have been found sufficiently accurate for screening preparation in low-resource environments.^14,15,16^ To reduce environmental noise, screening was scheduled for early mornings and after school hours.

The procedure of data collection was conducted in three phases:

- Preliminary consultations: Parents were engaged during annual school meetings to explain the purpose of the study and procedures, address concerns and ensure understanding and voluntary participation.
- Informed consent issuing: Parents were given informed consent forms to take home and completed whenever they were comfortable doing so and returned them to the school within two weeks for collection and analysis.
- Hearing screening: Screening followed HPCSA Minimum Standards for Hearing Screening in Schools (2018) and consisted of:
  ■ *Otoscopy*: This was the first step of the screening process. It is an examination of the external ear and auditory canal using a Welch-Allyn Mini 3000 otoscope to detect obstructions or foreign bodies.^9,10^ The learner does not have to respond during this objective procedure. If a foreign body, wax impaction, ear discharge, tympanic membrane perforation or redness is observed, the learner fails the otoscopic examination. If these are not observed, they proceed to the second step of screening. When they fail, they get referred to the local health facility for further management; when they pass, they proceed to tympanometry.
  ■ *Tympanometry*: The second step was the tympanometry. This is an objective assessment of middle ear function using a hand-held GSI 33 tympanometer.^10^ This test gives an interpretation in the form of Type A, which means there is no middle ear pathology; Type B, indicative of a middle ear pathology; Type C, indicative of negative or positive middle ear pressure; Type D, indicative of a flaccid tympanic membrane; Type Ad, indicative of low tympanic membrane impendance; and Type As, indicative of restricted tympanic membrane movement or high impendance. All types are abnormal except Type A. When the learner’s tympanometry results gave any other reading other than Type A, this was a failed hearing screening in step 2 because it means there is some form of middle ear pathology.^9,10^ When they pass tympanometry, they proceed to step 3, which is the pure-tone hearing screening; when they fail, they get referred to a local health facility.
  ■ *Screening audiometry*: This was the third and final step of the screening process. This is a subjective measurement of pure-tone thresholds at 500, 1000, 2000 and 4000 Hz using a GSI 33 audiometer, calibrated according to SABS standards. The learners were instructed to raise their hands whenever they heard a sound, whether it was soft or loud. The sound intensity was initiated at 40 dB for all learners and reduced by 5 dB steps until a threshold was reached. A pass decision was made when learners responded at 20 dB and below.^9,10^ When they pass this screening procedure, they pass the screening process, and when they fail, they are referred to the nearby health facility for further testing and diagnosis.
- Following the screening process, referrals were made as required, and communication of results was made to parents in writing.

### Data analysis

All hard copy data have been kept in a safe and locked office (the researcher’s office). All hearing screening data have been transferred and stored on a computer as well as a backup.^24^ The results have been consolidated on a Microsoft Excel spreadsheet and kept under a personal identification number (PIN) lock. The services of a statistician were used for data analysis. The data were analysed using the latest standardised statistical software, Statistical Package for Social Sciences (SPSS) version 27. The *t*-test has been calculated using statistical software. Thereafter, the *p*-value has also been investigated so that the researcher can conclude whether the study was significant with a *p*-value < 0.05. All results were interpreted and displayed as graphs, tables and charts.^25^

### Ethical considerations

Ethical clearance to conduct this study was obtained from the University of Fort Hare Health Research Ethics Committee (Ref#2022=11=10=Pierce A), and permission was granted by the Eastern Cape Department of Basic Education. The study followed the principles of the Declaration of Helsinki.^26^ Written parental consent and verbal assent from learners were obtained.

## Results

**FIGURE 1:**
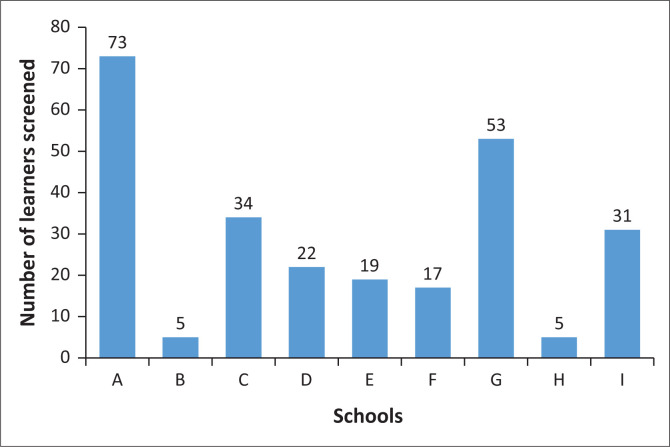
Distribution of screened learners by school.

These are results of the hearing screening, which aimed to determine the potential prevalence of hearing loss of Grade R learners in Qumbu CMC. The total number of schools recruited was 10; however, only 9 schools participated in the study. The 10th school could not be accessed because of the policy for conducting research in the last term of the year. Figure 1 shows the participation of learners per school. Table 1 shows the demographics of participants in terms of age, sex and race.

**TABLE 1:** Demographic characteristics of Grade R learners in Qumbu (*N* = 259).

Category	Subcategory	*n*	%
Age (years)	5	59	22.8
	6	200	77.2
Sex	Female	128	49.4
	Male	131	50.6
Race	Black people	259	100.0

The total number of participants was 259 out of a total of 481 learners from the 9 schools that participated in the research. This was a good turnout rate of 53.84%. Table 1 shows the demographic profile of participants.

Results show that out of the 259 participants, 66 (25%) failed the screening in at least one of their ears. The failing results applied at any stage of the screening (otoscopic examination, tympanometry or pure-tone screening audiometry). The remaining 193 (75%) learners passed the hearing screening. Passing meant that the learner had been to all three screening procedures and passed at all stages. The ones who had failed were referred to local health facilities for further diagnostic assessment and/or management. Refer to Table 2.

**TABLE 2:** Hearing screening results and cross-tabulation by gender.

Gender	Fail		Pass		Total	
	*n*	%	*n*	%	*N*	%
Female	35	27.34	93	72.66	128	100
Male	31	23.66	100	76.34	131	100

**Overall**	**66**	**25.48**	**193**	**74.52**	**259**	**100**

Note: Screening outcome: female: Fail - *n* = 35, 27.34%; Pass - *n* = 93, 72.66%; Total - *n* = 128, 100%. Screening outcome: male: Fail - *n* = 31, 23.66%; Pass - *n* = 100, 76.34%; Total - *n* = 131, 100%. Overall: Fail - *n* = 66, 25.48%; Pass - *n* = 193, 74.52%; Total - *N* = 259, 100.00%.

The investigation according to gender distribution consists of the 66 (25.5%) who failed the hearing screening test shows that, 35 (53%) were females and 31 (47%) were males. This suggests that the potential prevalence of hearing problems was higher among females than among males. To determine if this difference is statistically significant, the Chi-squared test for independence was carried out, and the results showed that there was no significant association between gender and hearing problems (Chi^2^ = 0.46; *p* = 0.496). The odds ratio for the association was found to be 1.2 with a 95% confidence interval of (0.69, 2.12). The confidence interval contains 1, the null value of odds ratios, which means the proportion of girls with hearing problems is not significantly different from that of boys.

**FIGURE 2:**
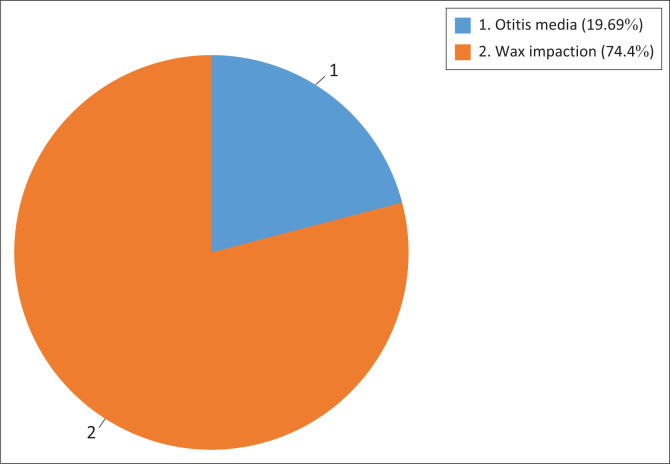
The reasons for failed hearing screening.

In terms of the causes of screening failure, the results refer to Figure 2. Figure 2 depicts the results from the screening of wax impact on learners as a risk factor.

Hearing screening results showed that 66 learner participants failed one of the hearing screening tests. Of the learner participants who failed the hearing screening, 13 (19.69%) presented with middle ear pathology (otitis media), and 49 (74.4%) presented with impacted wax. The learners who passed the otoscopic examination and middle ear pathologies have all passed the pure-tone hearing screening; hence, the pure-tone hearing screening results do not appear in the Figure 2.

## Discussion

The discussion is based on the results and objectives of the study. The first objective was to determine the referral rates and potential prevalence of hearing loss amongst 5–6-year-old Grade R learners in Qumbu CMC by conducting hearing screening.

The results of this study revealed a 25.5% referral rate, which needed further diagnostic assessment and management, which could confirm some prevalence of hearing loss among Grade R learners in Qumbu CMC or not. A majority of failures were caused by wax impaction, which causes temporal hearing loss. This is particularly concerning in a district with limited access to diagnostic services. Practically, it suggests that approximately 250 out of every 1000 Grade R learners may be living with an undetected cause of potential temporal or permanent hearing impairment. Given the importance of hearing in early childhood development, this figure indicates an urgent educational and developmental risk for the local population. The prevalence of impacted cerumen in Qumbu CMC was also notable. While often considered minor, wax impaction can cause temporary conductive hearing loss and interfere with screening accuracy. Govender et al. similarly identified cerumen impaction as a major factor in school hearing screenings in underserved South African areas.^27^ The WHO acknowledges wax impaction as a preventable cause of hearing loss.^9^ The high rate of wax impaction in Qumbu CMC may be attributed to anatomical factors, genetics or traditional cleaning practices – all cited in previous literature.^9,27^ In communities with poor access to ear care, these risk factors often go unmanaged, leading to preventable hearing impairment and developmental delays.

When compared to global benchmarks, the reported referral rates in Qumbu CMC are significantly higher. For example, a school-based screening in South India found hearing loss in only 11.9% of children aged 5–6 years,^28^ and a Polish study also reported a lower prevalence.^18^ In South Africa, results have varied: a Western Cape study identified unilateral hearing loss in 70% and bilateral in 30% of affected learners but did not report overall prevalence.^29^ A more recent study in Gauteng province reported a prevalence of just 2.2%.^29^ Such discrepancies may reflect different testing protocols, age ranges, environmental conditions and resource disparities.

If follow-up diagnostic testing were conducted, the actual prevalence might be lower because of the nature of screening tools and settings. The comparison of these screening results and existing literature increases the reliability of the findings.

The high referral rate and potential prevalence of hearing loss in Qumbu CMC are likely because of contextual factors such as inadequate access to healthcare, poor middle ear health management and low parental awareness. This region is in a similar context as a study done by Phanguphangu et al. in 2025, and these results concur with the results of social determinants of health and hearing loss in children.^13^ The results showed 19.67% of learners presented with middle ear pathologies. Although these were clustered and not further stratified in results, most of them were related to otitis media. Additionally, recurrent otitis media – a known risk factor – may contribute to transient or permanent hearing loss in rural children. These challenges highlight the necessity for structured school-based hearing screening supported by diagnostic services and public education campaigns. Without intervention, undetected hearing loss will continue to hinder school readiness and learning. This reinforces the need for routine, school-based screening programmes with diagnostic follow-up and educational support. Addressing these risks holistically is vital for preventing long-term communication and learning difficulties among children in rural districts such as Qumbu. Low screening rates and a lack of audiological infrastructure further exacerbate the issue. Without routine screening or parental concern, cases of mild to moderate hearing loss frequently go undetected until school age, when language and cognitive deficits may already be present. These findings underscore the need for integrated school-based hearing screening and targeted public health education to promote early detection and intervention. The results are consistent with similar studies where the wax impaction and middle ear pathologies, especially otitis media, were the most common causes of potential hearing loss in children of this age and similar context.

### Limitations of the study

The study was conducted in the period post-coronavirus disease 2019 (COVID-19) pandemic in 2022, and although at the time of data collection, the lockdown restrictions were lighter, some participants, especially parents, did not participate to avoid unknown consequences of mass data collection. Learners also took turns attending schools at the time, as a full-capacity class was not allowed. The context-relevant statistics on hearing loss within the Eastern Cape province were generally not available on the databases, and it was difficult to compare any similar studies. The data collection and results are only based on screening results, and no diagnostic follow-ups were conducted, which makes these results non-confirmatory of prevalence but only depicts a referral rate and a potential prevalence. This affects their generalisability, and inference to these findings should be avoided. This study can be used as a case example.

### Recommendations

It is recommended that structured, school-based hearing screening programmes, supported by diagnostic services and public education campaigns, be implemented. Without such interventions, undetected hearing loss will continue to compromise school readiness and learning.

Routine screening should be prioritised at school entry age and repeated once in every school phase, in line with policy requirements. These screenings should be conducted by trained school nurses and local clinic nurses to ensure accessibility and sustainability.

Health practitioners should also provide parents and teachers with information on ear hygiene, hearing health and the critical role of hearing in educational development.

A holistic approach that combines screening, diagnostic follow-up and education is essential to prevent long-term communication and learning difficulties, particularly among children in rural districts such as Qumbu.

## Conclusion

The hearing screening of Grade R learners aged 5–6 years in 2022 revealed a referral rate of 25.5% for wax impaction and middle ear pathologies. The primary objective of this study was to generate essential data to inform health policy and strengthen early hearing detection services for young children. This objective was achieved, and the study provides context-specific evidence that can guide future screening, research, policy development and management strategies for childhood hearing loss.
